# HBV Reactivation in Patients Undergoing Hematopoietic Stem Cell Transplantation: A Narrative Review

**DOI:** 10.3390/v11111049

**Published:** 2019-11-10

**Authors:** Giuseppe Gentile, Guido Antonelli

**Affiliations:** 1 Dept. Translational and Precision Medicine, Sapienza University of Rome, 00161 Rome, Italy; 2 Dept. Molecular Medicine, Sapienza University of Rome, 00161 Rome, Italy

**Keywords:** HBV reactivation, hepatitis B virus, hematopoietic stem transplantation, HSCT, HBV, chronic HBV infection, resolved HBV infection, occult HBV infection, immune escape

## Abstract

HBV reactivation (HBVr) can occur due to the ability of HBV to remain latent in the liver as covalently closed circular DNA and by the capacity of HBV to alter the immune system of the infected individuals. HBVr can occur in patients undergoing hematopoietic stem cell transplantation (HSCT) with a clinical spectrum that ranges from asymptomatic infection to fulminant hepatic failure. The risk of HBVr is determined by a complex interplay between host immunity, virus factors, and immunosuppression related to HSCT. All individuals who undergo HSCT should be screened for HBV. HSCT patients positive for HBsAg and also those HBcAb-positive/HBsAg-negative are at high risk of HBV reactivation (HBVr) due to profound and prolonged immunosuppression. Antiviral prophylaxis prevents HBVr, decreases HBVr-related morbidity and mortality in patients with chronic or previous HBV. The optimal duration of antiviral prophylaxis remains to be elucidated. The vaccination of HBV-naïve recipients and their donors against HBV prior to HSCT has an important role in the prevention of acquired HBV infection. This narrative review provides a comprehensive update on the current concepts, risk factors, molecular mechanisms, prevention, and management of HBVr in HSCT.

## 1. Introduction

Chronic hepatitis B virus (HBV) infection is a global public health issue, with the highest prevalence observed in Sub-Saharan Africa and East Asia, and with over 257 million people worldwide infected and with 887,000 deaths caused directly or indirectly by HBV every year [1]. Even in the non-endemic countries, the United States [2] and Europe [3], approximately 1 and 13 million individuals, respectively, have chronic HBV infection. Overall, approximately one-third of the world’s population have been infected and carry serological evidence of past or present HBV infection [4].

It is well known that patients with current (positive hepatitis B surface antigen (HBsAg)) or past (positive hepatitis B core antibody (HBcAb) and negative HBsAg) exposure to HBV infection who receive chemotherapy, immunosuppressive therapies, steroids or stem cell transplant may develop a HBV reactivation (HBVr) infection, potentially leading to interruption of chemotherapy and then adding significant morbidity and mortality [5]. In particular, patients undergoing allogeneic hematopoietic stem cell transplantation (allo-HSCT) are at a very high risk of HBVr, ranging from 14% to 78%, with a mortality rate of 5%–22% in patients without antiviral prophylaxis ([6] reviewed in [7]). Nevertheless, high frequencies of HBVr have also been documented in allo-HSCT with resolved HBV infection ranging from 2.6% to 42.9% [8,9,10,11]. The risk of HBVr in patients receiving autologous-HSCT (auto-HCT) is considered to be lower compared to allo-HSCT; however, in a study performed on HBsAg-positive auto-HSCT patients who were not receiving anti-HBV prophylaxis, HBVr was diagnosed in 50% of patients [12].

However, a standardized definition of HBVr has not been established. Heterogeneity of definitions for HBVr and its associated outcomes reported in the studies examining this topic may have underestimated the prevalence of HBVr, limiting the possibility to compare the results between studies.

It has been shown that the occurrence and the outcomes of HBVr are mainly related to two factors: immunity of the host and the characteristics of the HBV. Regarding the immunity of the patients with chronic HBV, it has been demonstrated, that the innate and adaptive immune response to the virus is not efficient, leading to the onset of chronic liver inflammatory events with subsequent development of cirrhosis and HCC [13]. In addition, the humoral response has a protective role in the host control of chronic or past infection [14], as shown by the frequent (risk of HBVr > 10%) HBVr observed in patients who have B-cell depletion as a result of treatment with monoclonal antibodies against CD20 (rituximab or ofatumumab) predominantly used to treat B-cell malignancies [5] and in the conditioning regimen of allo-HSCT (rituximab) [11]. Among the virologic factors, it is worth noting that HBsAg mutations may be associated with HBVr [15,16,17] and that the presence of immune-escape HBV mutations is often associated with impaired serological diagnosis of HBVr [18].

HSCT, previously known as bone marrow transplantation, has become the standard curative treatment for various onco-hematological malignancies (e.g., acute and chronic leukemia, multiple myeloma, lymphomas, and myeloproliferative neoplasms) and non-malignant diseases (e.g., aplastic anemia, myelodysplastic syndrome, immunodeficiency syndromes, genetic diseases, or hemoglobinopathies) [19]. The safety of HSCT has improved over the years and indications for HSCT have expanded to older patients [20]. These current conditions have contributed to an increasing number of HSCT survivors, which are estimated to be half a million worldwide [20]. Hepatic complications are a well known cause of post-HSCT morbidity and mortality [21]. Patients undergoing allo-HSCT usually have a higher risk for viral infections than auto-HSCT [19]. In this framework, it has been shown that in the setting of allo-HSCT, progression of the chronic viral infection or an increased risk of HBVr may be favoured by the severity and persistence of immunodeficiency observed in the post-transplantation period, usually due to an impaired immune reconstitution [22].

Given the high risk of HBVr in these patients, all HSCT patients should be screened for HBV infection [reviewed in 23]. Patients with current or prior exposure to HBV infection are candidates for prophylactic anti-HBV therapy whose duration is not yet clearly defined (reviewed in [23]). Universal prophylaxis with antivirals for anti-HBc-positive, HBsAg-negative HSCT recipients also remains controversial. The vaccination of HBV-naïve recipients and their donors against HBV prior to HSCT has an important role in the preventoin of acquired HBV infection. Donors of hematopoietic stem cells with current or past HBV infection can transmit viral hepatitis and should, therefore, be screened for HBV [23].

This narrative review provides a comprehensive update on the current concepts, risk factors, molecular mechanisms, prevention, and management of HBVr in HSCT.

## 2. Defining Hepatitis B Reactivation

Reactivation of HBV occurs in two distinct circumstances: in patients with chronic/inactive hepatitis B and in patients with resolved HBV infection (positive HBcAb and negative HBsAg) in whom the virus has apparently been cleared (so-called reverse seroconversion). The definitions of HBV reactivations are heterogeneous but predominantly include the following parameters [5,24]: (1) an acute rise in HBV DNA levels compared with baseline (or an absolute value of HBV DNA when a baseline level is not available); (2) elevated levels of serum aminotransferases; (3) reverse seroconversion (so called seroreversion), observed in patients HBsAg-negative/anti-HBc-positive/HBV DNA-negative who became HBsAg positive. Recently, the American Association for the Study of Liver Diseases (ASSLD) recommended new criteria for HBVr [24]. For patients who are HBsAg-positive, one of the following aspects should be considered: (1) ≥2 log (100-fold) increase in HBV DNA compared to the baseline; (2) HBV DNA ≥ 3 log (1000) IU/mL in a patient with previously undetected HBV DNA; or (3) HBV DNA ≥ 4 log (10000) IU/mL, if the baseline level is not available. For patients who are anti-HBc-positive and HBsAg-negative, the criteria that should be used are: (1) detection of HBV DNA or (2) occurrence of HBsAg “seroconversion” (reappearance of HBsAg).

The HBVr phase, from the virological point of wiew, e.g., viral replication, usually precedes hepatitis flare and as the patient’s immune function reconstitutes, symptoms and signs of hepatitis flare appear. Hepatitis flare is defined as an ALT increase to ≥3 times the baseline level and >100 U/L [24].

An area of controversy still exists about whether to consider clinical aspects other than serological profile and levels of viremia in the HBVr definition of the immunocompromised patients for onco-hematological malignancies. For example, it would be interesting to consider: (1) the degree of liver injury other than the hepatic inflammation (e.g., presence of icterus, ascites, coagulopathy and encephalopathy); (2) liver-related mortality; (3) consequences to underlying disease (interruption or discontinuation of immunosuppressive drugs). With the objective of reaching a uniform evaluation in reporting HBVr in immunocompromised patients among the studies, a scoring system that reports virologic, hepatic, and oncologic consequences has been proposed, but not yet validated [25].

## 3. Mechanisms of HBV Reactivation

The molecular biology of HBV, its replication, and mechanisms of immune control have been extensively reviewed [26,27,28,29].

### 3.1. HBV

In brief, HBV entry into the hepatocytes occurs by interaction with the key liver-specific receptor, sodium-taurocholate co-transporter [30]. Subsequently, the nucleocapsid is imported into the nucleus of hepatocytes, where DNA is converted in the form of the covalently closed circular DNA of the viral genome (cccDNA) [29]. The cccDNA is complexed with histones of the host and other cellular proteins to form a minichromosome as the template for viral transcription [31], which is regulated by epigenetic modifications and by transcriptional factors [32]. The cccDNA is stable in infected cells and can persist as a reservoir for HBVr [27]. A seminal study has shown that HBV DNA, presumably cccDNA and/or replicating HBV DNA, can persist in the liver of patients for decades after apparent recovery from HBV infection [33]. The persistence of cccDNA occurs despite active immune response and serologic clearance of HBV (loss of HBsAg and seroconversion to anti-HBs). In addition, clinical studies have shown that therapy with nucleos(t)ide analogues, such as adefovir dipivoxil or lamivudine, can suppress HBV DNA, with a modest reduction of cccDNA after one year of treatment [34,35]. Therefore, the clinical resolution of chronic or acute HBV infection does not result in the complete eradication of HBV, as cccDNA can persist in the liver of patients and may be a source of HBVr during immunosuppression [33,34].

HBV-induced hepatocellular damages are considered to be the results of a complex interplay among HBV, hepatocytes, and immune cells of the host and for this reason, several asymptomatic HBV carriers may have little or absent liver dysfunction, despite the presence of high HBV load [36,37].

The long-lasting persistence of replication-competent HBV cccDNA chromatinised episomes in hepatocytes associated with a strong suppression of viral replication and viral protein expression is the molecular basis for the occult hepatitis B infection (OBI) occurrence. OBI is defined as the presence of HBV DNA in the liver and/or blood (in the serum, it is usually very low, <200 IU/mL) of individuals who test negative for HBsAg by current commercial assays [38]. Based on HBV antibody profile, OBI may be classified as follows: (1) Seropositive OBI: presence of HBcAb and/or HBsAb; (2) Seronegative OBI: the absence of HBcAb and HBsAb. [36]. The main mechanisms of OBI remain unknown, but it is believed that several mechanisms might lead to the development of OBI. Among them there are (1) mutations in the preS1, preS2, and S regions of the HBsAg gene, which may result in undetectable HBsAg, (2) host epigenetic mechanisms (e.g., metilation of HBV DNA and histone alterations), (3) coinfections with HCV and HIV; (4) host factors such as such as immunologic and genetic alterations [38,39]. Patients with seropositive OBI may have apparently resolved acute HBV infection or chronic HBV infection with an apparent seroclearance of HBsAg but persistently low levels of transcription and viral replication [38,39]. Patients with OBI are at potential risk of HBVr during potent immunosuppressive therapies that may lead to life-threatening hepatitis for patients with onco-hematological diseases treated with anti-CD20 monoclonal antibodies (e.g., rituximab) or receiving HSCT [8,38,39,40].

### 3.2. Immunity

The course and the outcome of HBV infection are modulated by the host immune response and the loss of immune surveillance can cause HBV reactivation. HBV elicits a series of innate [41] and adaptive immune responses [42]. HBV clearance in adult immunocompetent individuals involves the induction of a strong polyclonal multispecific adaptive CD8+ T-cell response inducing both a cytolytic-dependent and -independent (suppression of viral replication by predominately interferons) antiviral effect by the expression of antiviral cytokines, as well as the induction of B-cell response with the production of neutralizing antibodies [43]. Neutralizing antibodies clear the circulating virus and limit the spread of HBV [42]. In addition, B cells act as potent antigen-presenting cells, particularly for helper T-cells [42]. Impairment in HBV-specific T-cell function favors the onset of chronic HBV infection [14,28]. It has also been shown that HBV evades host innate immunity to establish persistence [44,45]; HBV modifies Natural Killer (NK) cell receptors, suppresses NK cells to increase replication, control type I IFN responses, and leads to CD4 and CD8 exhaustion [44,45].

A peculiar characteristic of the HBV-host immune system interactions is that HBV can escape innate immune recognition. HBV might not induce an innate immune response because it is not detected by pattern recognition receptors, an effect known as the stealth properties of HBV [46]. It is interesting to note that HBV enhances rather than suppresses innate immunity within the infected hepatocytes [47]. For example, liver tissues from patients with chronic HBV infection do not have induction of an innate immune response, but this response can be activated by other factors (e.g., TLR3 binding) in HBV-infected liver tissue [48]. These findings support the hypothesis that HBV is invisible to pattern recognition receptors. In addition, HBV persistence is associated with the failure of T-cell control (reviewed in [27,28]). Chronic infection with HBV is characterized by exposure to high levels of virions and subviral particles and antigen presentation by hepatocytes play a key role in CD8+ T-cell exhaustion (reviewed in [27,28]).

The clinical relevance of adaptive immunity in efficient control of HBV infection mainly derives from studies in which transplantation of stem cells from HBV-immunized individuals, which contain HBV-specific memory T- and B-cells, into chronic HBV allo-HSCT patients, achieves control of chronic HBV infection [49,50,51].

Furthermore, low HBV-specific CD8+ T-cell counts and high B-cell IL-10 counts have been observed during antiviral therapy with entecavir in patients with HBVr after allo-HSCT [52]. In this study, the small decline in HBV DNA levels suggests an impaired therapeutic activity, despite 12 months of anti-HBV treatment, in patients without HBV-resistant strains [52].

Another clinically relevant issue on adaptive immunity in allo-HSCT comes from studies showing that recipient-derived humoral immunity seems to have a protective role against HBVr after allo-HSCT [53]. However, the loss of previous HBV immunity might be inevitable as a result of the loss of the recipient’s immune cells in combination with the prolonged inadequate T-cell-dependent B-cell responses [54].

### 3.3. Immunoppressive Drugs

HBVr that occurs in HSCT may be associated with the use of immunosuppressive drugs that broadly target host functions and are used for prophylaxis or treatment of graft-versus-host-disease (GVHD). For example, cyclosporin, an immunophilin inhibitor, suppresses T-lymphocyte activity [55] and the use of high-dose steroids suppresses cell-mediated immunity by inhibiting the production of interleukins relevant for T- and B-cell proliferation [56]. In addition, steroids directly modify HBV replication through the glucocorticosteroid-responsive element in the HBV genome, leading to upregulation of HBV gene expression [57]. Other immunosuppressive agents, such as monoclonal antibodies rituximab [11] and alemtuzumab [58], which are used as a part of conditioning regimen of HSCT might be associated with a risk of HBVr. In particular, rituximab targets the B-cell surface antigen CD20, inhibits B-cell activation, causes a long-term effect of B-cell depletion, which generally lasts for several months after therapy [5,11,58]. Furthermore, rituximab impacts the immune response by modulating B-/T-cell interactions, possibly changing the dynamic balance between the host immune response to HBV and the degree of viral replication. In addition, B-cell depletion exerts a deleterious impact on the induction, maintenance and activation of cell-mediated immunity (reviewed in [59]). Alemtuzumab, an anti-CD52 monoclonal antibody, induces severe depletion of peripheral blood lymphocytes (both T- and B-cells, especially CD4 T-cells), and this effect is profound and long-lasting. CD52 is expressed on most lymphocytes, monocytes, macrophages, and epithelial cells. Several cases of HBVr after alemtuzumab therapy have been reported ([53], and reviewed in [59]).

## 4. Clinical Outcomes of HBV Reactivation

The clinical spectrum of HBVr of HSCT patients appears heterogeneous with respect to its frequency, manifestation, and outcome. HBVr can be subclinical [60] and resolve spontaneously, or result in clinically apparent acute hepatitis, which can be severe—evolving into acute liver failure and death [61]. In addition, long-term complications, such as liver cirrhosis, can occur at a frequency of about 10% after a median follow-up of 7 years [62].

However, other causes of liver derangement in HSCT include drug-induced liver damage, other hepatotrophic (HCV and HEV) and nonhepatotrophic viruses (Epstein–Barr virus, cytomegalovirus or herpes simplex virus); GVHD, sinusoidal hepatitis obstruction syndrome, obliterative portal venopathy, or autoimmune hepatitis should be excluded [21].

The time of onset of HBVr varies from an average of 10 to 48 months post allo-HSCT and a median of 16 months from auto-HSCT [40,60,63,64]. However, retrospective studies performed on patients with resolved HBV and receiving allo-HSCT showed that HBVr can even develop up to 5 years following transplantation because of a long period of delay for reconstitution of the recipient’s immune response to HBV [9,65]. It is likely that several cases of reactivation are unnoticed, as studies have shown that about 25% of HBVr cases that have virological criteria for reactivation do not show an increase in transaminase levels [5].

Following HBVr, liver-related mortality has been evaluated in large studies; some have shown a mortality rate of >50% in immunocompromised patients, whereas others have shown liver-related mortality ranging from 0% to 20% of cases [5,66,67].

As regards HSCT patients, few studies have focused their analysis on mortality due to HBV-related events. Retrospective studies carried out in the 1990s showed that death due to severe liver failure occurred in 21% of allo-HSCT patients with chronic hepatitis B (CHB) [68]. Subsequently, a case-control study carried out in the 2000s showed that only preemptive lamivudine therapy effectively reduced mortality due to exacerbation of HBV hepatitis in HBsAg-positive allo-HSCT patients [69]. Intriguingly, more recent retrospective studies performed in allo- and auto-HSCT with resolved HBV infection found that HBVr did not adversely affect patient survival (e.g., progression-free survival and overall survival) [11,64,70].

## 5. Interplay of Risk Factors for HBV Reactivation in HSCT

The main risk factors for HBVr in general belong to three main categories: (1) host factors, (2) virologic factors, and (3) type and degree of immunosuppression.

### 5.1. Host Risk Factors

The male sex has been shown to be a risk factor for HBV reactivation in cancer patients. Yeo et al., [71] found a three-times higher incidence in men than in women in 626 cancer-positive HBsAg patients who underwent chemotherapy, although the reason for this finding is not clear.

It has been shown that age ≥50 years was an independent risk factor for HBVr in patients with resolved HBV infection undergoing allo-HSCT [60,72]. Age-related immune senescence may contribute to HBV reactivation or another possible explanation is that anti-HBc-positive, HBsAg-negative patients ≥50 years of age, could have had CHB that had reached HBsAg seroclearance, with the mean age of HBsAg seroclearance estimated to be around 50 years of age [73]. These patients have HBV persisting at low trascriptional and replicative levels and are, therefore, more likely to have HBVr with suppression of immunity during HSCT. It is interesting to note that immunocompetent patients with CHB who achieve HBsAg seroclearance generally have a favourable clinical outcome. However, there is a low yet definite risk of hepatocellular carcinoma occurrence, particularly in male CHB patients who achieve HBsAg seroclearance when they are over 50 years old (reviewed in [39]).

The presence of cirrhosis in patients with hematological malignancies and resolved HBV infection undergoing allo-HSCT has been shown to be an independent risk factor for HBVr [74].

### 5.2. Virologic Factors for HSCT

They include detectable HBV DNA prior to transplantation (high baseline HBV DNA level), hepatitis B e antigen positivity, and chronic hepatitis B. Other virologic factors that predispose one to HBVr in HSCT include the absence of anti-HBs in either HSCT recipients [9] or donors [11]. Recent studies have shown that patients with lymphoma treated with rituximab, with high HBcAb levels and low HBsAb levels, have a high risk of HBVr [75,76].

As regards HSCT recipients, two retrospective studies [40,65] showed that high anti-HBc titers (7.5 S/CO and 8 S/CO, respectively) have been identified as risk factors for HBVr. A possible explanation is that high anti-HBc titers might correlate with a hepatic reservoir of HBV, which decreases after seroclearance and could potentially explain the association of high anti-HBc titer with HBVr. In contrast, Seto et al. [60], in a prospective study, showed that anti-HBs status evaluated before starting HSCT and serial modifications after transplantation in anti-HBs levels were not associated with HBVr.

Specific HBV genotypes have been associated with more rapid progression and severity of hepatitis B in immunocompetent patients (reviewed in [77]); a clear role regarding the association of HBV genotypes with HBVr is still unknown and is, in our opinion, a relevant issue to address.

HBV mutations have been reported to be a probable factor associated with HBVr in immuncompromised patients, such as those receiving HSCT (reviewed in [17] and [18]. In a recent, retrospective study, Anastasiou OE et al. [40] found that 13 of 55 (23.6%) allo-HSCT patients developed HBVr. The HBV genome was sequenced from serum samples of eight patients with HBVr and in four of them, immune escape variants were detected either as majority or minority variants. Interestingly, studies have been reported that immunosuppression could favor the production of mutated viral species with increased potential to evade immune responses [15,16]. Whether variants with modified S protein emerge after viral reactivation or whether patients with past HBV infection harbouring those variants are at risk for HBVr is still unknown.

### 5.3. Type and Degree of Immunodepression

Patients undergoing HSCT typically receive intense chemotherapy for the underlying malignancy to induce remission, followed by additional chemotherapy and radiation chemotherapy to ablate bone marrow [58]. Patients undergoing allo-HSCT requiring more intense and prolonged immunosuppression have a higher risk of HBVr than auto-HSCT recipients [65]. Patients with GVHD (acute and/or chronic) are at risk for HBVr since they are further immunocompromised by the routine use of prophylactic cyclosporine and methotrexate or mycophenolate mofetil for the prevention of GVHD [53,60]. When GVHD is diagnosed, treatment with high doses of steroids or antithymocyte globulin or both further suppress the host immunity [58]. In addition, GVHD delays proper reconstitution of the immune system for up to 12–18 months [78]. Furthermore, the duration of immunosuppression is also a risk for HBVr in HSCT recipients [8,11].

Infection due to cytomegalovirus (CMV) is also immunosuppressive and its role as risk factor for HBVr has not been evaluated. Following primary infection, CMV establishes lifelong latency; reactivation from latency in allo-HSCT patients is clinically expressed through direct and indirect effects (reviewed in [79]). Direct effects are clinical manifestations due to CMV replication (e.g., pneumonitis, gastrointestinal diseases, hepatitis, marrow suppression, retinitis, and CNS diseases) whereas indirect effects based on CMV-immunodulatory activities may lead to an increase in the incidence of other opportunistic infections, an increase of the risk for acute and chronic organ rejection, and a decrease in patient survival (reviewed in [79]). The co-infection between HBV and CMV in an HSCT setting has been little investigated [80,81,82]. In the allo-HSCT setting, a study found an association between HBsAg seropositivity and increased risk of CMV DNAemia [82], while another study did not show any relation between HBsAg serostatus and the incidence of CMV reactivation [81]. Differences between the results of these studies might be related to different study populations, different methods of diagnosis of CMV reactivation, different conditioning regimens, immunosuppressive agents and/or other unknown factors.

As regards auto-HSCT, an Italian study showed an association between positivity for anti-HBc and CMV reactivation [80]. It has been shown that HBV/CMV infection is associated with expansion of memory-like NK cells of immunocompetent patients with chronic HBV infection/CMV infection, revealing that NK-cell repertoire is mutually affected by CMV and HBV coinfection [83]. This study underlines the concept that HBV/CMV co-infection can shape the immune repertoire and affects the immune response in patients with chronic HBV [83,84].

In allo-HSCT, the immunologic reconstitution plays an important role on the onset of acute GVHD and CMV infection; in particular, a high content of Tregs appears to protect from the occurrence of both phenomena, as well as a high content of NK cells for CMV infection [85]. On this basis, future studies should evaluate whether HBV plays a role in triggering CMV reactivation or if the failure of HBV clearance is a potential consequence of CMV reactivation in HSCT recipients. It is worth noting that the issue on the association between HBVr and CMV infection might also apply to other viruses, even if this issue is totally unknown.

## 6. Management of HBV Reactivation

### 6.1. Screening

All patients who are candidates to receive an HSCT should be screened for HBV before starting HSCT. However, there are no prospective randomized studies on HBV screening to establish the advantages of one screening approach over another. Based on the current knowledge, international guidelines recommend that HSCT recipients be tested for HBsAg, HBcAb, and HBsAb [23,24,86]. Based on the serology results, an evaluation of quantitative HBV DNA is recommended, which can be used as a baseline for follow-up [23,24,86]. This strategy may overlook the patient with occult HBV who has a low titer of HBV DNA, negative HBsAg and positive anti-HBc. The Italian guidelines [23], developed for patients with haematologic malignancies and patients who underwent haematologic stem cell transplantation recommend that quantitative HBV DNA be tested for patients who HBsAg-positive/anti-HBc-positive or HBsAg-negative/anti-HBc-positve with or without anti-HBs positivity.

### 6.2. Prophylaxis of HBV Reactivation in Allogeneic Hematopoietic Stem Cell Transplantation in Patients with Chronic or Past HBV Infection

#### 6.2.1. Prophylaxis of HBV Reactivation in Allo-HSCT Recipients with Chronic HBV Infection

Patients undergoing allo-HSCT are at a very high risk of HBVr, with a HBsAg reactivation rate of up to 45% in patients without antiviral prophylaxis and up to 23% in patients receiving antiviral prophylaxis with lamivudine in case-control or retrospective studies with historical control (reviewed in [7]). Concerning the use of antiviral prophylaxis in HBsAg-positive allo-HSCT recipients, the probability of survival without hepatitis owing to exacerbation of HBV was significantly lower in HBsAg-positive patients receiving lamivudine than in those without prophylaxis (54.3% vs. 94.1%) [69]. In this study, HSCT recipients receiving lamivudine also had a significantly higher cumulative incidence of sustained HBsAg clearance compared with HSCT recipients without prophylaxis [69]. Overall, few retrospective and prospective studies have shown that lamivudine and entecavir are both effective for reducing the HBVr in HBsAg-positive allo-HSCT recipients [87,88]. A recent retrospective study with historical control compared the efficacy of lamivudine vs. entecavir in 216 HBsAg-positive allo-HSCT recipients [89]. The cumulative incidence of HBVr at 6, 12 and 24 months following transplantation were 3%, 7%, and 24% in the lamivudine group and 0%, 0%, and 2% in the entecavir group, respectively. Entecavir was associated with lower cumulative incidence of severe hepatitis associated to HBVr than lamivudine. Drug resistance mutations were observed in 25 patients in the lamivudine group vs. only 1 patient in the entecavir group [89]. HBsAg seroclearance was seen similar in both groups of patients. Overall, all the patients who achieved HBsAg seroclearance remained HBsAg-negative and HBV DNA was undetectable to the end of the follow-up period; none developed HBV reactivation.

A recent systematic review with meta-analysis evaluated the efficacy of prophylaxis for HBVr after allo-HSCT in the era of drug resistance and the availability of newer antivirals [90]. Data from 10 studies in which 611 allo-HSCT recipients (either HBsAg-positive or anti-HBc-positive/HBsAg negative patients) received anti-HBV prophylaxis show that the aggregate HBVr rate was 1.9% in patients receiving entecavir and 11.5% in the group receiving lamivudine; no studies were available that used tenofovir, telbivudine, or adefovir for HBvr prophylaxis in allo-HSCT [90]. To our knowledge, only one case report of two allo-HSCT patients with GHVD who successfully received tenofovir for HBVr is available [91]. Antiviral drugs should be started at least one week before the HSCT procedure and should be continued for at least one year [7]. As regards the duration of lamivudine or entecavir prophylaxis, it ranged between 6 and 30 months in the evaluated studies [90]. Ideally, the duration of antiviral prophylaxis should be based on immune recovery, which can take years following allo-HSCT, but it is reasonable to consider lifelong antiviral treatment in these high-risk patients [92]. To our knowledge, no studies have been published regarding the optimal duration and the type of antiviral treatment in this type of patients. Therefore, on the basis of the available data, all HBsAg-positive HSCT recipients should be treated independently of the presence or the level of HBV DNA [23,24,86,93] with a high-genetic-barrier antiviral drug (entecavir, tenofovir, or tenofovir alafenamide fumarate) [23,24,83] and for at least one year or more pending immune reconstitution of the HSCT recipients. Close monitoring (every 1–3 months) of viremic rebound and sero-reversion should be performed when prophylaxis is discontinued [7,23,94]. The timing of monitoring is not actually defined. The disappearance of HBV DNA and HBsAg associated with the appearance of anti-HBs are indicative of resolved infection and allow the suspension of antiviral therapy.

#### 6.2.2. Prophylaxis of HBV Reactivation in Allo-HSCT Recipients with Past HBV Infection

The frequency of HBVr after allo-HSCT in HBsAg-negative, anti-HBcAb-positive patients, ranges from 2.6% to 42.9% in retrospective studies [8,9,10,11,95,96]. In a recent prospective study performed on Asian allo-HSCT patients, the cumulative rate of HBVr was 40.8% [60]. These variable frequencies of HBVr among published studies might be associated with the selection bias, different definitions of HBVr, different frequencies and methods of HBV DNA monitoring, the lack of regular monitoring after HSCT or using HBsAg seroreversion, a relatively late clinical event compared to the increase of HBV DNA, to define HBVr [8,9,95].

Universal prophylactic anti-HBV therapy can prevent HBVr in HBsAg-negative, anti-HBc-positive patients undergoing HSCT [91,97,98,99]. However, in allo- and auto-HSCT with resolved HBV infection, a retrospective cohort study with a control group demonstrated that a short-term (median 7 months) of lamivudine prophylaxis was not sufficient to prevent HBV reactivation in a median time of 20 months after HSCT [70]. In this regard, universal prophylactic with anti-HBV therapy in HBsAg-negative, anti-HBc-positive patients undergoing HSCT remains controversial. It is recommended in several [7,23,24,86,94] but not in all guidelines [100,101]. Nucleos(t)ide analogue(s) prophylaxis in anti-HBc-positive HSCT recipients is recommended by the European Association for the Study of the Liver (EASL) [86], by ASSLD [24] and by the Italian guidelines [7,23]. However, ASSLD [24] and EASL [86] consider entecavir, tenofovir (tenofovir disoproxil fumarate), or tenofovir alafenamide fumarate (TAF) as the choice for prophylaxis because all three drugs have a high genetic resistance, while EASL [86] and the Italian guidelines [23] recommend the use of lamivudine in this setting for a time period of at least 18 months. To date, no studies have been published regarding the optimal duration and the type of antiviral treatment in this type of patient. On the contrary, in a recent study, Seto et al. [60] prospectively followed HBsAg-negative, anti-HBc-positive allo-HSCT recipients by performing, every 4 weeks, HBV-DNA monitoring until week 104 post-HSCT, and than every 3 months. At the time of HBVr, defined as HBVDNA >10 UI/mL, patients received entecavir with suppression of HBV DNA to undetectable levels and without any clinical hepatic events. However, this study showed that allo-HSCT aged >50 years, with or without GVHD, were at high risk of HBVr (61.8% within 2 years); therefore, the authors suggest that, in centers without resources to perform frequent HBV DNA monitoring, prophylactic anti-HBV therapy should be given to allo-HSCT patients [60]. Further studies are needed to compare the efficacy and feasibility of the universal prophylactic antiviral approach versus on-demand anti-HBV therapy in HBsAg-negative, anti-HBc-positive HSCT recipients.

#### 6.2.3. Management of HBV Infection in Allo-hsct Donor

In the setting of allo-HSCT, an additional risk of HBVr is related to the donor HBV status. In a case-control study, a high incidence of hepatitis related to HBVr was associated with marrow in fusion from a HBsAg-positive donor [102] and transmission rates were significantly lower in anti-HBs-positive patients. The development of HBV-related hepatitis was associated with a donor with precore/core promoter mutations compared with donors with other HBV variants [102]. In a prospective study with a historical control group [103], the use of antiviral drugs, of anti-HBV immunoglobulin, and vaccination appeared to protect the HSCT recipient from HBV-related hepatitis and hepatic failure.

Very little is known about the condition of HBV-negative allo-HSCT recipients with anti-HBc-positive+/−anti-HBs-positive donors. Only retrospective reports have documented the absence of HBV transmission in adult HBV-naive HSCT recipients treated with lamivudine while receiving HSCT from an anti-HBc-positive donor [98,104]. A protective role of HBV immune/exposed HBV donors in anti-HBc-positive HSCT recipients has been reported in two retrospective studies [11,95]. All HBV-negative allo-HSCT recipients with anti-HBc-positive/anti-HBs-positive or -negative (HBV-DNA-negative) donors should receive prophylaxis with lamivudine [23]. The lamivudine prophylaxis duration is not defined.

### 6.3. Prophylaxis of HBV Reactivation in Autologous Hematopoietic Stem Cell Transplantation Patients with Chronic or Past HBV Infection

The risk of HBVr in patients receiving auto-HCT is considered lower compared to allo-HSCT. However, in HBsAg-positive auto-HSCT patients who were not receiving anti-HBV prophylaxis, HBVr was shown in 50% of patients [12]. In two retrospective studies performed in multiple myeloma patients who received a sequential bortezomib-containing induction therapy and auto-HSCT, it was found that the positivity for HBsAg was an independent risk factor for overall survival [105,106]. A nationwide retrospective study in Japan showed that auto-HSCT was an independent risk factor for HBVr in patients with resolved HBV infection [107]. The cumulative incidence of HBVr in patients with auto-HSCT at 2 and 5 years was 16% and 30.6%, respectively [107]. A multivariate analysis revealed that auto-HSCT was independently associated with a high prevalence of HBVr; in contrast, lenalidomide significantly decreased the incidence of HBVr. Due to the small number of studies available, the current guidelines do not differentiate between autologous and allogeneic HSCT regarding the risk for HBVr and its management.

### 6.4. HBV Vaccination

All patients undergoing HSCT who are HBV-negative at screening and patients who were vaccinated before HSCT but lost their protective immunity 6 months after transplant should undergo HBV vaccination 6–12 months after HSCT [108] and their anti-HBs titre should be periodically monitored [7,23,108]. A standard vaccination schedule (20 mg at 0, 4 and 6 months) is generally recommended [7,23,108]. However, about 50% of the HSCT recipients lose seroprotection within 6 months [109] from the time of transplant and 90% of the patients were unprotected after 5 years [110]. In a retrospective study with a historical control group, none of the anti-HBc-positive HSCT patients in the vaccine group developed post-HSCT HBV seroreversion [111].

Since an anti-HBc donor can transmit HBV to the recipient, even in the absence of detectable HBV DNA [112], the European Conference on Infections in Leukaemia (ECIL 7) [110] recommend vaccinating the recipients before HSCT. HBV vaccination of HBV-negative auto- or allo-HSCT patients should be performed before beginning the conditioning regimen. In the allo-HSCT setting, vaccination of donors for HBV-positive recipients is also suggested, with the goal to elicit a specific adaptive immune response that protects the recipient from HBV reactivation [113]. In patients who were previously infected with HBV, in addition to the use of anti-HBV drugs, vaccination might prevent reverse seroconversion [114]. The ECIL 7 recommends the evaluation of anti-HBc titres 6 months after transplant, and 1–2 months post-vaccination; it advises that a second series of vaccinations be considered in those patients with anti-HBs < 10 mIU/mL, even if the benefit has not been clearly proven [108].

## 7. Conclusions

HBVr is not uncommon in HSCT recipients and it is associated with a relevant morbidity, or even mortality; therefore, it requires appropriate management. The key issue to prevent HBVr is the identification of patients at risk of reactivation and this can be obtained by performing a careful screening for HBV prior to the onset of HSCT. It is important to assess the risk for HBVr, which is given from the interplay of factors such as the clinical history, the virologic characteristic of HBV, and the grade of immunosuppression induced by HSCT procedures, with the objective of determining the need for antiviral prophylaxis and its relative duration. Areas of unmet needs include a better stratification of HBVr risk, especially in HSCT patients with resolved HBV infection. Furthermore, there is a need of viral markers that better reflect the dynamics of cccDNA and HBV replication.
